# Adrenomedullin increases cAMP accumulation and BDNF expression in rat DRG and spinal motor neurons 

**DOI:** 10.22038/ijbms.2021.54796.12289

**Published:** 2021-07

**Authors:** Mohsen Sisakht, Zahra Khoshdel, Amir Mahmoodazdeh, Sayed Mohammad Shafiee, Mohammad Ali Takhshid

**Affiliations:** 1 Department of Biochemistry, School of Medicine, Shiraz University of Medical Sciences, Shiraz, Iran; 2 Department of Medical Laboratory Sciences, School of Paramedical Sciences, Shiraz University of Medical Sciences, Shiraz, Iran; 3 Diagnostic Laboratory Sciences and Technology Research Center, School of Paramedical Sciences, Shiraz University of Medical Sciences, Shiraz, Iran

**Keywords:** Adrenomedullin, Cyclic AMP, Nerve growth factors, Signaling transduction, Spinal cord

## Abstract

**Objective(s):**

Adrenomedullin (AM) has high expression in the spinal cord. In this study, we investigated the expression of AM and its receptor components, including calcitonin receptor-like receptor (CLR) and receptor activity modifying proteins (RAMPs) in dorsal root ganglion (DRG) and spinal motor (SM) neurons. Furthermore, the effects of AM on cAMP/cAMP response element-binding protein (CREB), AKT/glycogen synthase kinase-3 beta (GSK-3β) signaling pathways, and expressions of brain-derived neurotrophic factor (BDNF) and neurotrophin-3 (NT-3) were evaluated.

**Materials and Methods:**

Rat embryonic DRG and SM neurons were isolated, purified, and cultured. Real-time PCR was used to assess expressions of AM, CLR, and RAMPs. cAMP levels, p-CREB, BDNF, and NT-3 were determined using an enzyme-linked immunosorbent assay. p-AKT and p-GSK-3β levels were determined by western blotting. Real-time PCR showed expressions of AM, CLR, RAMP2, and RAMP3 in both DRG and SM neurons.

**Results:**

AM increased cAMP accumulation and p-CREB levels in DRG and SM neurons. AM increased p-AKT and p-GSK-3β in DRG, but not SM neurons. AM significantly increased BDNF expression in both DRG and SM neurons. There was also an increase in NT-3 level in both DRG and SM neurons, which is statistically significant in SM neurons.

**Conclusion:**

These results showed both DRG and SM neurons are targets of AM actions in the spinal cord. An increase in BDNF expression by AM in both DRG and SM neurons suggests the possible beneficial role of AM in protecting, survival, and regeneration of sensory and motor neurons.

## Introduction

Adrenomedullin (AM) is a peptide with 52 amino acids, known initially as a vasodilator peptide (1). However, further investigation showed that AM had high expression in various parts of the brain and spinal cord (2). In the spinal cord, AM expression occurs in the dorsal root ganglion (DRG) and dorsal horn, both neuronal and glial cells (2, 3). The bioactivity of AM is mainly mediated through AM1 and AM2 receptors, heterodimeric receptors consist of calcitonin receptor-like receptor (CLR) and one of the receptor-activity-modifying proteins RAMP-2 or -3 (4). Both AM receptors, particularly AM1 (CLR/RAMP-2) are sensitive to the antagonistic effects of AM_22–52_ (5). Also, expression of both CLR and RAMPs was revealed in rat DRG and spinal cord (6-8). Animal studies have shown increased AM-like immunoreactivity in the DRG and spinal cord following several pathological conditions including spinal cord injuries (9), formalin- (10), capsaicin-, complete Freund’s adjuvant-induced inflammatory pain (2), and repeated intrathecal (i.t.) injection of morphine (11), suggestive of the pronociceptive role of AM in both mechanical and chemical-induced pain (12). cAMP/protein kinase A (PKA)/phosphorylated cAMP response element-binding protein (p-CREB) is the most characterized AM signaling pathway (5). In many cell lines and primary cells, including dissociated spinal cells (13), stimulatory effects of AM on cAMP accumulation and p-CREB were shown. AKT/GSK3 is another signaling pathway with an important role in mediating AM effects. Ma *et al.* showed an increased level of phosphorylated AKT(p-AKT) and -GSK-3β (p-GSKβ3) in the superficial layer of rat spinal dorsal horn, following intrathecal AM administration, suggesting the role of AKT/GSK3 pathway in AM-induced hyperalgesia (8). Furthermore, in the cultured rat cardiomyocytes, AM overexpression reduced ischemia/reperfusion-induced apoptosis through AKT/GSK-3β signaling pathway (14). 

Brain-derived neurotrophic factor (BDNF) and Neurotrophin 3 (NT-3) are members of the neurotrophins family, essential for the growth, differentiation, and survival of neurons via activating tropomyosin receptor kinase receptors. BDNF is normally expressed in DRG neurons as well as sensory and motor neurons of the spinal cord. BDNF expression increases in the DRG and sensory neurons in response to various nociceptive stimuli, suggesting its role in pain processing (15). Motor neurons are the final component of neuronal circuits that transmit signals from the CNS to muscles (16). Animal studies revealed the critical role of BDNF in the functional properties and plasticity of motor neurons (17). The importance of NT-3 has been highlighted in axonal regeneration, especially in the spinal cord (18, 19) and brain injuries (20). BDNF and NT-3 are downstream targets of cAMP/PKA/CREB, and their gene expression increases in response to the increased cellular accumulation of cAMP (21, 22).

To the best of our knowledge, no study has addressed the expression of AM and its receptor components, including CLR and RAMPs in the motor neurons of the spinal cord, yet. Herein, we evaluated and compared the expression of AM, CLR, and RAMP2-3 in dissociated rat DRG and motor neurons. We also investigated the effects of AM on the activation of cAMP/CREB and AKT/GSK-3β pathways in both DRG and motor neurons and the expression of BDNF and NT-3. 

## Materials and Methods

***Chemicals***

Rat AM and AM 22-52 were obtained from Bachem Americas, Inc. (Torrance, CA, USA). All chemicals and western blot materials used in this study were obtained from Sigma-Aldrich, Santa Cruz, and Abcam. ELISA kit was obtained from Promega.

***Animals***

Female Sprague-Dawley rats, weighing 200–250 g (n=120), were provided by the Laboratory Animal Center of Shiraz University of Medical Sciences, Shiraz, Iran. Rats were mated, and detection of the vaginal plaque was considered the first day of pregnancy. Rats were housed 4 to a cage in transparent cages (59×38×20 cm) and received water and food *ad libitum* under a 12 hr light/dark cycle. On day 14 rats were decapitated after CO_2_ inhalation. All experimental protocols were performed in accordance with the National Institutes of Health Guide for Care and Use of Laboratory Animals and were approved by the Medical and Research Ethics Committee of the Shiraz University of Medical Sciences, Shiraz, Iran. 

***Isolation and culture of embryonic rat DRG and SM neurons***

**Figure 1:**
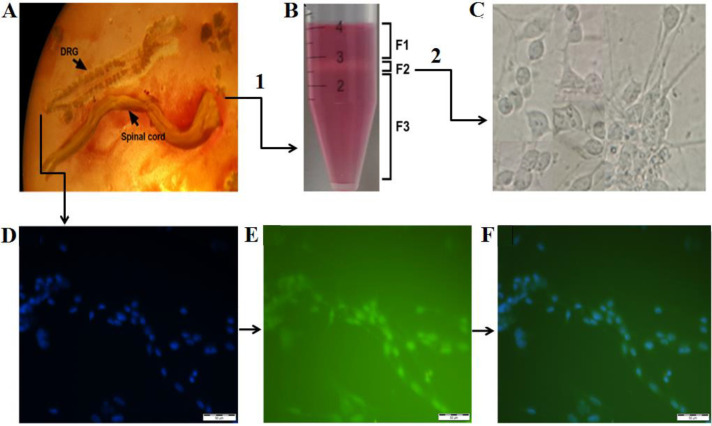
Dorsal root ganglion (DRG) and spinal motor neuron cells isolated from rat embryo. Figure A shows the spinal cord and the associated DRGs. (B) OptiPrep gradient was used to separate motor neurons. F1, F2, and F3 layers represent cell debris, motor neurons, and non-neural cells (capsule, fibroblasts, and Schwann cells), respectively. Figure C shows the purified spinal motor neuron's culture, (D) DAPI stained DRG cells, (E) Anti-β III-tubulin positive DRG neurons, (F) merged image of D and E (200X). Scale bars: 50 µm

Embryonic rat DRG neurons culture was prepared according to the method described by Burkey *et al*. (23). Briefly, Sprague-Dawley rat embryos on day 15 of gestation (E15) were dislocated, and the DRGs were separated (Figure 1). The DRGs were gently digested using trypsin (0.125%, Sigma-Aldrich, Saint Louis, MO, USA) and mechanical trituration for 15 min at 37 °C. The resulting cell suspension was seeded on poly-L-lysine coated plates (48 well culture plate) and cultured at Neuro Basal-A medium supplemented with 2% B27 containing 2 mM GlutaMAX, 100 mg streptomycin, 100 units of penicillin, at 37 ^°^C with 5% CO_2_ and 80% relative humidity. The cell density was adjusted to 1x 10^5^ cells ml^-1^. To remove non-neural cells, the day after incubation, cells were treated with 5-fluorodeoxyuridine (FUDR) and uridine at a final concentration of 20 mM for 72 hr. Anti-β III-tubulin (neuronal marker) monoclonal fluorescent antibody and DAPI staining were used to determine the purity of the DRG neurons (24). Briefly, the cells were placed on a coverslip and treated with ice-cold 3.7% formaldehyde in PBS for 10 min. The cells were then washed with 0.5% bovine serum albumin (BSA) solution, blocked by 5% normal goat serum supplemented with 0.5% BSA solution (1 hr at room temperature), and incubated with β -III-tubulin mouse monoclonal antibody (Cell Signaling, diluted (1:1000) in a mixture of 0.5% BSA and 0.01% Triton X-100) for 1 hr at room temperature. Goat anti-mouse secondary antibody conjugated with Alexa 488 (Cell Signaling, diluted (1:100) in the dilution buffer) along with 1 µg/ml DAPI (Sigma) was then added. Incubation was continued for 30 min in the dark at room temperature. Finally, the cells were washed and visualized under a fluorescent microscope. 

**Figure 2:**
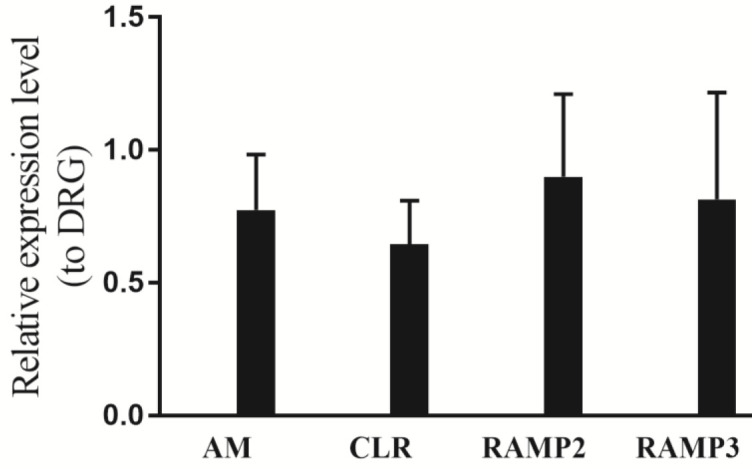
Expression of adrenimedullin (AM), calcitonin receptor-like receptor (CLR) receptor-activity-modifying protein 2 (RAMP2), receptor-activity-modifying protein 3 (RAMP3) in the spinal motor neurons in comparison with dorsal root ganglion (DRG) neurons. Bars represent means; error bars represent SEM of at least six independent experiments. Expression levels of AM, CLR, and RAMP-2 and -3 transcripts were normalized with β-actin and analyzed by ∆∆CT method. Student’s t-test was performed for statistical analysis of data

SM neurons were isolated, purified, and cultured, according to Wang *et al*. (25). Briefly, spinal cords were digested in 0.025% trypsin (Invitrogen), and spinal cells were dissociated using trituration. Separating solution (12.4% of OptiPrep in DMEM) was added to the spinal cell suspension and centrifuged at 2200 rpm (10 min at 4 ^°^C). Three distinct cellular layers (upper: cell debris, interface: motor neurons, and lower: non-neural cells) were separated (Figure 1). The motor neuron layer was carefully separated, seeded on poly-L-lysine coated plates (48 well culture plate) and cultured in Neuro Basal-A medium supplemented with 2% B27, containing 2 mM GlutaMAX, 100 mg streptomycin, 100 units of penicillin at 37 ^°^C with 5% CO_2_ and 80% relative humidity. The cell density was adjusted to 1x 10^5^ cells ml^-1^. The day after incubation, cells were treated with 5-fluorodeoxyuridine (FUDR) and uridine at a final concentration of 20 mM for 72 hr (26) to remove non-neural cells (Figure 2). 

***Quantitative real-time PCR (qRT- PCR)***

The expression of AM, CLR, RAMP2, and RAMP3 was measured using quantitative real-time PCR. Briefly, total RNA from DRG and SM neurons were extracted, using BIOZOL-RNA; RNA extraction reagent (BSC51M1, Zhejiang, China), and the cDNA was synthesized by a cDNA Synthesis Kit (Fermentase, USA), using the manufacturers’ instructions. SYBR Green Real-time PCR (Applied Biosystems, USA) was conducted to determine gene expressions of AM, CLR, RAMP2, RAMP3, and β-Actin, using specific primers (Table 1). Each gene’s relative expression was normalized respective of the endogenous control, β-Actin, and calculated with the 2-ΔΔCt formula. Differences in C_t_ values of two genes were calculated as the percentage or fold difference compared with control.

***cAMP assay***

To evaluate AM’s effect on cAMP accumulation (27), DRG and SM neurons were cultured in serum-free DMEM containing 1 μM RO-20-1724(4-(3-Butoxy-4-methoxybenzyl)-2-imidazolidinone; an inhibitor of cAMP phosphodiesterase for 30 min. The cells were incubated with AM (10 ^−11^ to 10^− 6^ M) in the presence or absence of AM receptor antagonist (AM 22-52) for 10 min before terminating the reaction by adding ice-cold ethanol (100%). The cells treated with adenylyl cyclase activator, forskolin (1 μM), served as the positive controls. The cAMP level was measured by a specific ELISA kit (Cayman chemical, Item No. 581001) and in accordance with the manufacturer’s instructions.

***Quantification of phosphorylated-CREB (p-CREB) in DRG and SM neurons***

The aforementioned protocol (i.e., cAMP assay) was used to determine AM’s effect on the level of p-CREB/CREB. Briefly, DRG and spinal cells were incubated with AM (25 nM) for 20 min. The p-CREB (Ser133) level was assayed in the cell lysate by DuoSet IC Phospho-CREB (S133) ELISA kit (R&D Systems). 

***Western blot***

Western blotting was used to detect AKT (Santa Cruz Biotechnology, Santa Cruz, CA, USA), p-AKT (Ser 473) (Cell Signaling, 9271), GSK-3β (Cell Signaling, 9315), and p-GSK-3β (Ser 9) (Cell Signaling, 9322). β-Tubulin (Cell Signaling, 4466) was used for normalization. The protocol was conducted as previously reported (28). NP40 lysis buffer (20 mM Tris-HCl (pH 7.5), 0.5% Nonidet P-40, 0.5 mM PMSF, 100 μM β-glycerol 3-phosphate, protease inhibitor cocktails (Sigma, Cat#: P8340), and phosphatase inhibitor) was used to extract proteins. Protein concentration was quantified by the BCA assay kit (Novagen, San Diego CA, USA). Following heating to 90 ^°^C for 5 min, the proteins (20 µg) were separated by 12.5% SDS–polyacrylamide gel electrophoresis (SDS-PAGE) and transferred to nitrocellulose membranes. Non-fat dry milk (5%) and TBST (Tris-buffered saline containing 0.01% Tween 20) were used as blocking buffer (1 hr at room temperature). Incubation with primary antibodies was performed overnight at 4 ºC. HRP-conjugated secondary antibody (St. Louis, MO, USA) was applied for 2 hr at room temperature. Washing with 1×TBST was performed before adding primary and secondary antibodies. Enhanced chemiluminescence (ECL) reagent (ab133406) was finally added to the membranes for 5 min. The ChemiDoc TM MP System ((Bio-Rad, USA) was used to visualize the bands. Protein expression was estimated by the dosimetry software Image Lab (Bio-Rad).

***Enzyme-linked immunosorbent assay***

BDNF and NT3 concentrations were measured using ELISA kits (Cloud-Clone USA, and Elabscience USA, respectively). Total protein was measured by the Bradford method. Results were normalized to the total protein concentration.

***Statistical analysis***

Graph pad Prism program (Version 6, GraphPad Software, San Diego, CA, USA) and SPSS software (version 21) were used for statistical analysis. Data are presented as mean ± SEM. Differences between groups were assessed by ANOVA followed by the Student-Newman-Keuls *post hoc* analysis. Differences were considered significant at *P*<0.05. pro-BDNF protein expression’s densitometric data were analyzed using the Mann Whitney U test, and *P*<0.05 was considered a significant difference between the groups. EC_50_ antagonist/EC_50_ agonist was calculated by plotting sigmoidal concentration-response curves. The pA2 values were calculated by log[antagonist] - log(EC_50_ antagonist/EC_50_ agonist - 1).

## Results

***Purification and culture of DRG and spinal motor neurons***

Figure 1 shows isolation, purification, and culture steps of rat embryonic SM (A, B, C) and DRG (D, E, F) neurons**. **To prepare DRG neurons’ culture, DRGs were isolated from rat embryos (Figure 1-A), digested, and treated with FUDR to remove non-neuronal cells. The results of Anti-β III-tubulin staining (Figure 1-E) revealed that 92±7 % of the cultured cells were DRG neurons. A gradient of OptiPrep™ was applied to purify the SM neurons. Figure 1 B shows three distinct cellular layers (F1: cell debris, F2: SM neurons, and F3: non-neural cells) were observed after centrifugation. F2 layer containing SM neurons was removed and cultured. Evaluation of the cultured cells morphology by phase-contrast microscopy (Figure 1 C) revealed that 87±5% of the cultured cells were SM neurons.

***Expression of AM, CLR, RAMP-2, and RAMP-3 in DRG and spinal motor neurons***

Real-time PCR was used to evaluate the expression of AM and its receptor components (CLR and RAMP-2 and -3) in DRG and SM neurons. The data revealed expressions of AM, CLR, and RAMP-2 and -3 in both neurons (Figure 2). No significant difference was observed in AM’s expression and its receptor components between the DRG and SM neurons. 

***Characterization of AM receptors using the cAMP assay***

**Figure 3:**
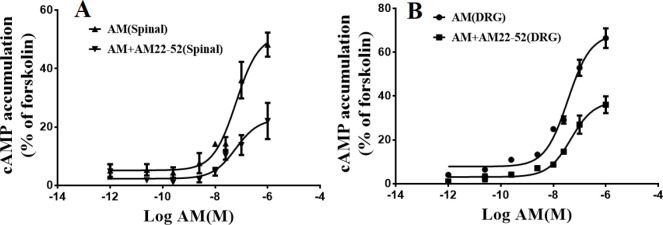
Cellular accumulation of cAMP was increased in rat embryonic spinal motor neurons(A) and dorsal root ganglion (DRG) neurons(B) following treatment with different adrenomedullin (AM) concentrations. The data are mean±SEM of at least three independent experiments and are expressed as the percentage of maximum cAMP accumulation. The maximum cAMP level was estimated by fitting each line in a logistic Hill equation

**Figure 4:**
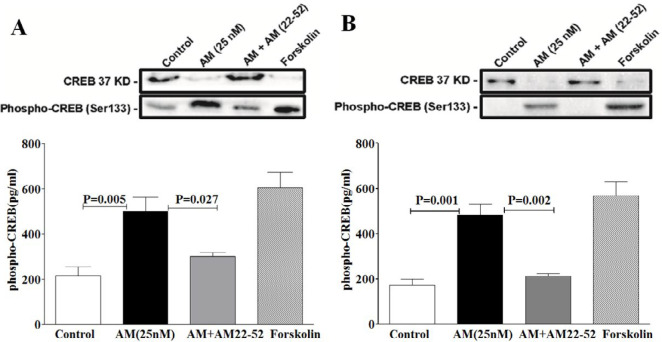
Level of phosphorylated-cAMP response element-binding protein (p-CREB) was increased in dorsal root ganglion (DRG) (A) and spinal motor neurons (B) following treatment with adrenomedullin (AM;25 nM). ELISA was used to measure the level of p-CREB (Ser133) in both DRG (A) and spinal motor neurons (B) following exposure to AM and forskolin. Pretreatment with AM22-52 reversed the AM-induced p-CREB elevation in both DRG (A) and spinal motor neurons (B). The data were analyzed using one way-ANOVA, followed by Dunnett's post hoc tests. Data are presented as mean±SEM, and at least three independent experiments were performed

Since there is a well-known association between AM receptors and the cAMP/p-CREB signaling pathway, AM’s effects on the cellular accumulation of cAMP and p-CREB were evaluated. Our data revealed that forskolin (1 μM), an adenylyl cyclase activator, significantly elevated cAMP accumulation in both DRG (54.04±0.7) and SM neurons (53.62±45). AM increased cAMP accumulation dose-dependently in both DRG (EC_50_=85.76 nM) and SM neurons (EC_50_=103.3 nM). The peak of cAMP levels was observed at 1 μM AM, which were 16.6 and 9.5 times higher than the basal levels in DRG and SM neurons, respectively (Figure 3). The p-CREB level was also elevated following DRG (Figure 4 A) and motor neuron exposure to forskolin (Figure 4 B). Pretreatment with AM receptor antagonist (AM 22-52) inhibited AM-induced cAMP accumulation in both DRG and SM neurons with pA2 values of 7.59 and 7.62, respectively. Following treatment with AM (25 nM), the level of p-CREB increased by 2.35 and 2.8 times compared with baseline in the DRG (Figure 4 A) and SM neurons, respectively (Figure 4 B). Pretreatment with AM 22-52 inhibited AM-induced p-CREB elevation in both DRG (Figure 4 A) and SM neurons (Figure 4 B). These data suggest possible role of the cAMP/p-CREB signaling pathway in AM-induced activities in both DRG and motor spinal neurons. 

***p-AKT and p-GSK-3β levels in AM-exposed DRG and spinal motor neurons***

**Figure 5:**
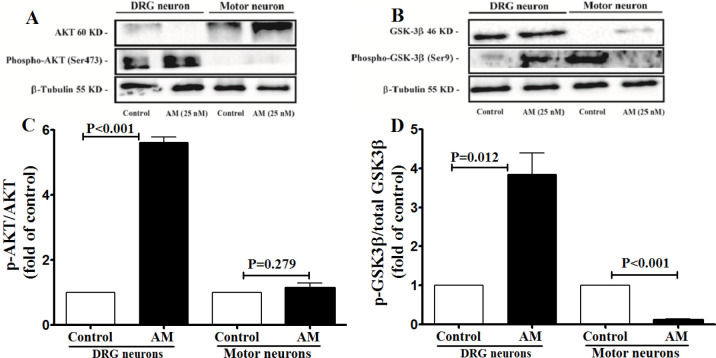
Adrenomedullin (AM ; 25 nM) effects on the ratio of p-AKT/AKT and phosphorylated-glycogen synthase kinase-3 beta (p-GSK-3β) /GSK-3β in the dorsal root ganglion (DRG) and spinal motor neurons. A and B show the results of western blot analysis of p-AKT/AKT and p-GSK-3β/GSK-3β, respectively. C and D show the relative levels of p-AKT/AKT and p-GSK-3β/GSK-3β, respectively. The data were analyzed by student’s t-test and presented as mean±SEM. The experiments were repeated three times with similar results

Western blot analysis showed a significant increase in the level of p-AKT (Ser473) (Figure 5 A) and p-GSK-3β (Figure 5 B) in DRG neurons treated with AM (25 nM) compared with the controls. In the SM neurons, no significant alteration was observed in the p-AKT (Ser473) level following treating with AM (25 nM) compared with the controls (Figure 5 B). 

***The effects of AM on BDNF and NT3 expression in DRG and spinal motor neurons***

**Figure 6:**
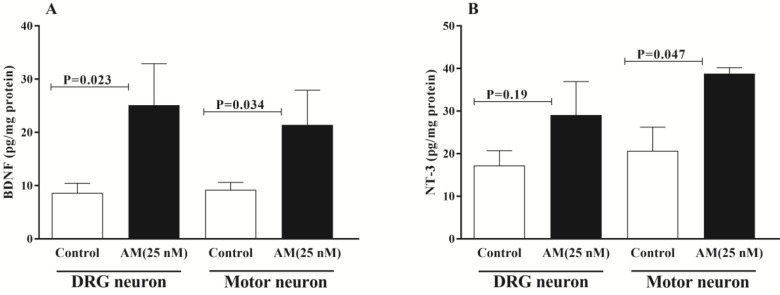
Adrenomedullin (AM, 25 nM) increased the brain-derived neurotrophic factor (BDNF) level in DRG and spinal motor neurons cells (A). AM also significantly increased neurotrophin 3 (NT-3) levels in the SM neurons. The significance of each group’s differences was analyzed by student’s t-test with P<0.05 as the level of significance. Bars represent mean, error bars represent SEM of at least three independent experiments

To investigate the effects of AM on expression of BDNF and NT3, DRG and SM neurons were treated with AM (25 nM), and the levels of these proteins were measured using specific ELISA methods. The result of ELISA showed a significant increase in the BDNF protein level in both DRG and SM neurons after treatment with AM (25 nM) (Figure 6 A). There was also an increase in the NT-3 level in both DRG and spinal motor neurons, which is statistically significant only in the latter case following exposure to 25 nM AM (Figure 6B). 

**Table 1:** Primer sequences used in real-time PCR

**Name**	**Primer sequence (5'3')**	**Size (bp)**
AM	Forward:GAACAACTCCAGCCTTTACC Reverse:GAGCGAACCCAATAACATCAG	62
CLR	Forward:CACACCAAGCAGAATCCAATC Reverse:GTCATACACCTCCTCAGCAA	59.3
RAMP2	Forward:GAATCAATCTCATCCTACT Reverse:TGTAATACCTGCTAATCAA	56.3
RAMP3	Forward:CTGACCTCTGCTACGCTTG Reverse:TGACTCCTAACAACTCCATTCC	62.2

AM: Adrenomedullin; CLR: Calcitonin receptor-like receptor; RAMP2: Receptor-activity-modifying protein 2; RAMP-3: Receptor-activity-modifying protein 3

## Discussion

In the present study, the data revealed expression of AM and its receptor components, CLR and RAMP-2 and -3, in both DRG and spinal motor neurons. Dose dependently, AM increased cAMP accumulation in both DRG (EC_50_=85.76 nM) and SM neurons (EC_50_=103.3 nM) in a manner that was sensitive to antagonistic effects of AM22-52, suggesting receptor-mediated AM effects. Furthermore, the p-CREB level increased in response to AM treatment, suggesting the cAMP/CREB signaling pathway’s possible role in AM-mediated effects in both DRG and SM neurons. An increase in phosphorylated AKT/GSK-3β following AM treatment suggests that this pathway might also be involved in AM effects in DRG neurons but not in SM neurons. The expression of BDNF increased in both DRG and SM neurons following treatment with AM. As far as we know, this is the first report on AM expression and function in the SM neurons. 

Our data revealed expression of AM and its receptor components, CLR and RAMP-2 and -3, in rat DRG neurons, which are in line with the previous studies (6, 11). Our findings also revealed expression of AM, CLR, and RAMP-2 and -3 in the SM neurons. To our knowledge, this is the first report on expression of AM and its specific receptor components in the SM neurons, which suggests that motor neurons are new targets for the AM effects. Furthermore, the effects of AM on induction of signaling pathways involved in the growth, development, and repair of motor neurons, as well as increased BDNF expression suggest that AM may play beneficial roles in protecting motor neurons against neurodegenerative diseases that target SM neurons.

 The cAMP/p-CREB signaling pathway in mediating AM affects the peripheral tissues (29, 30) and various parts of the CNS (13, 31) is well documented. We evaluated AM’s effects on the cAMP accumulation in both DRG and SM neurons to characterize AM receptors. In agreement with the previous studies (13, 32), our results revealed that AM increases cAMP accumulation (EC_50_=85.76 nM) and p-CREB level in the DRG neurons. Similarly, AM increased cAMP accumulation (EC_50_=103.3 nM) and p-CREB level in the SM neurons. These effects were antagonized by AM22-52, a selective antagonist for AM receptors (33), suggesting a receptor-mediated AM effect in both neurons. 

It was reported that activation of the AKT/GSK-3β signaling pathway contributes to the protective effects of AM against apoptosis induced by several conditions, such as hypoxia-induced apoptosis in mesenchymal stem cells (34), lipopolysaccharide-induced apoptosis in rat Leydig cells (35), mannitol-induced apoptosis in human umbilical vein endothelial cells (36), and ischemia/reperfusion-induced apoptosis in myocardial cells (14). AKT is a serine/threonine kinase, activated through phosphorylation by phosphoinositide-dependent kinase 1 (37). p-AKT can trigger phosphorylation and inhibit GSK-3β. The results of western blot analysis revealed significant effect of AM on the increasing level of p-AKT and p-GSK-3β levels in the DRG neurons, which agrees with the AM effects in other cells (34, 35). In the mouse model of neuropathic pain, blocking GSK-3β, using its specific inhibitor (AR-A014418), reduced proinflammatory cytokine production, suggesting its role in the inflammatory neuropathic pain (38, 39). We have recently shown that AM protects DRG against doxorubicin-induced cell death (40); hence, the AKT/GSK-3β signaling pathway might contribute to this effect. The GSK-3β is also involved in neuroinflammation (41), neurodegeneration (42), neuronal extension (43), myelin destruction (44), and also pathogenesis of immune-mediated diseases of the neuronal system (45). In addition, in rat models of cerebral ischemia, transferring the AM gene to neuron induced anti-apoptotic effects, shown by the overexpression of Bcl-2, p-AKT, and p-GSK-3β (46). The increase in the level of AKT/GSK-3β in AM-treated DRG neurons signifies this signaling pathway’s potential role in AM-induced survival of DRG neurons. However, this phosphorylation pathway seems to be insignificant in AM-induced effects in the SM neurons. There are several possible explanations for these findings. First, it is well known that AM can activate different signaling pathways by acting on one type of receptor and induces different physiological effects in various cell types. Second, our data showed different EC_50_ for AM in increasing cAMP accumulation in DRG (EC_50_=85.76 nM) and SM neurons (EC_50_=103.3 nM), suggesting the presence of various kinds of receptors with different affinities for AM in DRG and SM neurons. Finally, although the expression of AM receptor components was not significantly different at the mRNA level in the DRG and SM neurons, however, the expression of these components may differently be regulated at the protein and post-translational levels in the DRG and SM neurons, leading to formation of AM receptors with various affinities that activate various signaling pathways. 

BNDF and NT3 are normally expressed in both DRG and SM neurons (47, 48). Our results revealed a significant increase in BDNF concentration in both DRG and SM neurons following AM treatment. Although the exact underlying mechanism was not investigated, previous studies showed that BDNF expression increased by the cAMP/CREB pathway (49, 50); hence, up-regulation of BDNF by AM might result from activation of the cAMP/ CREB pathway. The role of BNDF in many aspects of spinal sensory and motor activities, including cell survival, growth, differentiation, and regeneration, is well documented (48, 51, 52). Our recent studies showed the protective role of AM against doxorubicin-induced cell death in both DRG(40) and motor neurons (not published data), whether AM plays a role in these BDNF-induced activities, especially nerve regeneration requires further investigation.

## Conclusion

Our findings revealed expression of AM and its receptor components in both DRG and SM neurons. Moreover, AM increased cAMP and p-CREB accumulation in both DRG and SM neurons. AM also induced phosphorylation and inactivation of GSK-3β in DRG, but not SM neurons. Finally, AM induced BDNF expression in both DRG and spinal motor neurons. Considering the known activities of BDNF, AM can serve as a protective and surviving factor for both DRG and SM neurons.
